# Prevalence of Gene Rearrangements in Mexican Children with Acute Lymphoblastic Leukemia: A Population Study—Report from the Mexican Interinstitutional Group for the Identification of the Causes of Childhood Leukemia

**DOI:** 10.1155/2014/210560

**Published:** 2014-07-17

**Authors:** Vilma Carolina Bekker-Méndez, Enrique Miranda-Peralta, Juan Carlos Núñez-Enríquez, Irma Olarte-Carrillo, Francisco Xavier Guerra-Castillo, Ericka Nelly Pompa-Mera, Alicia Ocaña-Mondragón, Angélica Rangel-López, Roberto Bernáldez-Ríos, Aurora Medina-Sanson, Elva Jiménez-Hernández, Raquel Amador-Sánchez, José Gabriel Peñaloza-González, José de Diego Flores-Chapa, Arturo Fajardo-Gutiérrez, Janet Flores-Lujano, María del Carmen Rodríguez-Zepeda, Elisa María Dorantes-Acosta, Victoria Bolea-Murga, Nancy Núñez-Villegas, Martha Margarita Velázquez-Aviña, José Refugio Torres-Nava, Nancy Carolina Reyes-Zepeda, Cesar González-Bonilla, Juan Manuel Mejía-Aranguré

**Affiliations:** ^1^Unidad de Investigación Médica en Inmunología e Infectología, Hospital de Infectología “Dr. Daniel Méndez Hernández”, “La Raza”, Instituto Mexicano del Seguro Social (IMSS), Avenida Jacarandas Esquina Vallejo S/N colonia La Raza, 02990 México, DF, Mexico; ^2^Laboratorio de Biología Molecular, Hospital General de México, Secretaria de Salud (SS), Eje 2A Sur (Dr. Balmis) 148, Col. Doctores, Delegación Cuauhtémoc, 06726 México, DF, Mexico; ^3^Unidad de Investigación Médica en Epidemiología Clínica, Hospital de Pediatría, Centro Médico Nacional (CMN) “Siglo XXI”, Instituto Mexicano del Seguro Social (IMSS), Avenida Cuauhtémoc 330, Delegación Cuauhtémoc, 06720 México, DF, Mexico; ^4^Servicio de Hematología, UMAE Hospital de Pediatría, Centro Médico Nacional (CMN) “Siglo XXI”, Instituto Mexicano del Seguro Social (IMSS), Avenida Cuauhtémoc 330, Delegación Cuauhtémoc, 06720 México, DF, Mexico; ^5^Departamento de Hemato-Oncología, Hospital Infantil de México Federico Gómez, Secretaria de Salud (SS), Calle Doctor Márquez 162, Colonia Doctores, Delegación Cuauhtémoc, 06720 México, DF, Mexico; ^6^Servicio de Hematología Pediátrica, Hospital General “Gaudencio González Garza”, Centro Médico Nacional (CMN) “La Raza”, Instituto Mexicano del Seguro Social (IMSS), Calzada Vallejo y Jacarandas S/N Colonia La Raza, Delegación Azcapotzalco, 02990 México, DF, Mexico; ^7^Servicio de Hematología Pediátrica, Hospital General Regional “Carlos McGregor Sánchez Navarro”, Instituto Mexicano del Seguro Social (IMSS), Avenida Gabriel Mancera No. 222, Colonia Del Valle, 03100 México, DF, Mexico; ^8^Servicio de Onco-Pediatría, Hospital Juárez de México, Secretaria de Salud (SS), Avenida Instituto Politécnico Nacional 5160, Colonia Magdalena de las Salinas, Delegación Gustavo A. Madero, 07760 México, DF, Mexico; ^9^Servicio de Hematología Pediátrica, CMN “20 de Noviembre”, Instituto de Seguridad Social al Servicio de los Trabajadores del Estado (ISSSTE), Félix Cuevas 540, Colonia Del Valle, Delegación Benito Juárez, 03229 México, DF, Mexico; ^10^Servicio de Hematología Pediátrica, Hospital General de México, Secretaria de Salud (SS), Eje 2A Sur (Dr. Balmis) 148, Col. Doctores, Delegación Cuauhtémoc, 06726 México, DF, Mexico; ^11^Servicio de Oncología Pediátrica, Hospital Pediátrico de Moctezuma, Secretaria de Salud del DF (SSDF), Oriente 158-189, Colonia Moctezuma 2a Sección, Delegación Venustiano Carranza, 15530 México, DF, Mexico; ^12^Laboratorio de la Coordinación de Vigilancia Epidemiológica y Apoyo en Contingencias, Unidad de Investigación Médica en Inmunología e Infectología, Hospital de Infectología “Dr. Daniel Méndez Hernández”, “La Raza”, Instituto Mexicano del Seguro Social (IMSS), Calzada Vallejo y Jacarandas S/N Colonia La Raza, Delegación Azcapotzalco, 02990 México, DF, Mexico; ^13^Coordinación de Investigación en Salud, Instituto Mexicano del Seguro Social (IMSS), Avenida Cuauhtémoc 330, 4to Piso Edificio de la Academia Nacional de Medicina, 06720 México, DF, Mexico

## Abstract

Mexico has one of the highest incidences of childhood leukemia worldwide and significantly higher mortality rates for this disease compared with other countries. One possible cause is the high prevalence of gene rearrangements associated with the etiology or with a poor prognosis of childhood acute lymphoblastic leukemia (ALL). The aims of this multicenter study were to determine the prevalence of the four most common gene rearrangements [*ETV6-RUNX1, TCF3-PBX1, BCR-ABL1*, and *MLL* rearrangements] and to explore their relationship with mortality rates during the first year of treatment in ALL children from Mexico City. Patients were recruited from eight public hospitals during 2010–2012. A total of 282 bone marrow samples were obtained at each child's diagnosis for screening by conventional and multiplex reverse transcription polymerase chain reaction to determine the gene rearrangements. Gene rearrangements were detected in 50 (17.7%) patients. *ETV6-RUNX1* was detected in 21 (7.4%) patients, *TCF3-PBX1* in 20 (7.1%) patients, *BCR-ABL1* in 5 (1.8%) patients, and *MLL* rearrangements in 4 (1.4%) patients. The earliest deaths occurred at months 1, 2, and 3 after diagnosis in patients with *MLL, ETV6-RUNX1*, and *BCR-ABL1* gene rearrangements, respectively. Gene rearrangements could be related to the aggressiveness of leukemia observed in Mexican children.

## 1. Introduction 

Leukemia is the most common cancer in children worldwide, and acute lymphoblastic leukemia (ALL) is the most common subtype, accounting for 80% of all cases [1]. Mexico has two major problems in relation to childhood leukemia: it has one of the highest incidences of childhood leukemia in the world [2], and it has significantly higher mortality rates for this disease compared with other countries [3].

The related factors for these two problems in Mexico are not completely understood. However, it has been suggested that one factor could be the high prevalence of gene rearrangements associated with the etiology or with a poor prognosis of children with ALL [4, 5].

In Mexico City, three studies have reported the frequencies of gene rearrangements in children with ALL. They were single hospital studies based on a small number of cases [4–6]. Pérez-Vera et al. [4] reported a low frequency of the* ETV6-RUNX1* gene rearrangement in 57 Mexican patients with leukemia from the Instituto Nacional de Pediatría. In another study by Jiménez-Morales et al. [5], a high proportion of* TCF3-PBX1* cases was reported in 53 ALL patients. Finally, Daniel-Cravioto et al. [6], in one of the hospitals at the Instituto Mexicano del Seguro Social (IMSS), reported a high frequency of the* MLL-AF4* gene rearrangement, which has been associated with leukemia of a poor prognosis.

When a child is diagnosed with leukemia in Mexico City, the detection of gene rearrangements is not routinely performed. Therefore, the prognostic stratification and the choice of chemotherapy treatment are based on clinical characteristics, laboratory tests, and the immunophenotype [7].

There is no available population level information on the prevalence of gene rearrangements in Mexican patients with childhood ALL.

The aims of this multicenter study were to determine the prevalence of the four most common gene rearrangements [*ETV6*-*RUNX1*,* TCF3*-*PBX1*,* BCR*-*ABL1*, and* MLL* rearrangements] and to explore their relationship with mortality rates during the first year of treatment in ALL children from Mexico City.

## 2. Materials and Methods

### 2.1. Patients

The Mexican Interinstitutional Group for the Identification of the Causes of Childhood Leukemia (MIGICCL) conducted a prospective study of newly diagnosed ALL patients below the age of 19 years between January 1, 2010, and December 31, 2012, in eight public hospitals in Mexico City. The diagnosis of ALL was based on bone marrow morphology and immunophenotyping. Patients were treated with existing local treatment protocols, which differ from one hospital to another. The present study was approved by the National Ethics and Scientific Committees with the following number: 2009-785-001. Informed consent was obtained from the children's parents in accordance with the Declaration of Helsinki.

### 2.2. Hospitals

**Figure 1:**
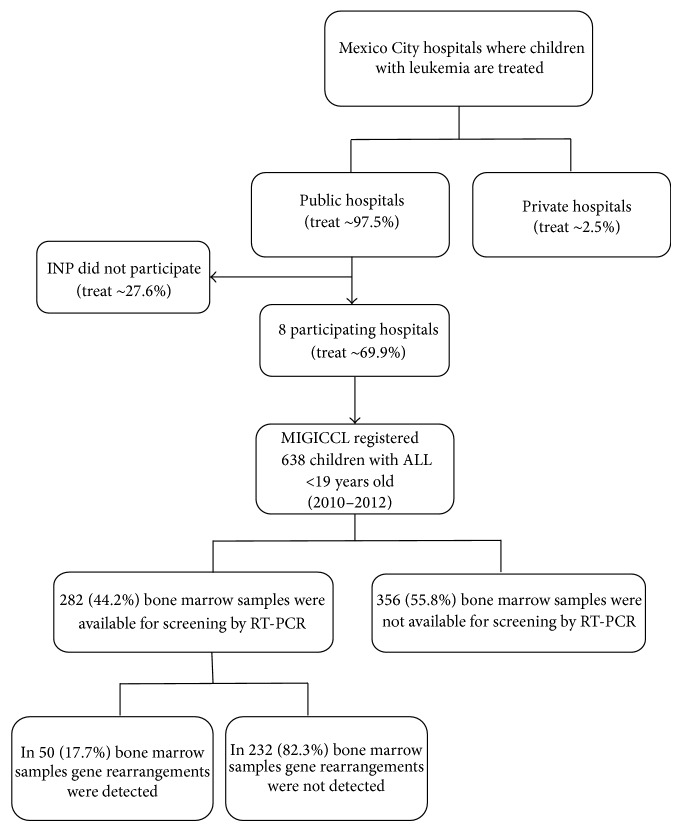
Flow chart of the selection process. Mexican Interinstitutional Group for the Identification of the Causes of Childhood Leukemia (MIGICCL) study of newly diagnosed ALL patients below the age of 19 years between January 1, 2010, and December 31, 2012, in eight public hospitals in Mexico City.

It has been estimated that the majority (97.5%) of children with leukemia are treated in nine public hospitals of Mexico City (Figure 1). The remaining cases are treated at private institutions [2]. The Instituto Nacional de Pediatría (INP) did not participate in this study because approval from the INP Institutional Review Board was not granted.

Participating hospitals represent four different Mexican Health Institutions: the Hospital de Pediatría, Centro Médico Nacional (CMN) “Siglo XXI,” the Hospital General “Gaudencio González Garza,” CMN “La Raza” and the Hospital General Regional “Carlos McGregor Sánchez Navarro” from the Instituto Mexicano del Seguro Social (IMSS), the Hospital Infantil de México Federico Gómez, the Hospital General de México and the Hospital Juárez de México from the Secretaria de Salud (SSa), the Hospital Pediátrico de Moctezuma from the Secretaría de Salud del Distrito Federal (SSDF), and the Hospital CMN “20 de Noviembre” from the Instituto de Seguridad Social al Servicio de los Trabajadores del Estado (ISSSTE).

### 2.3. Clinical Data and Definitions

The following clinical data were collected from patient records: information regarding sex, age at diagnosis, immunophenotype classification, white blood cell (WBC) count at diagnosis, and date of the patient's last visit to the hospital or the date of the patient's death as of one year after ALL diagnosis.

According to the National Cancer Institute (NCI) risk classification, patients were classified as having a standard risk [with ages ranging from 1 to 9.99 years and initial white blood cell count (WBC) < 50 × 10^9^/L)] or a high risk [age <1 or ≥10 years and/or initial WBC ≥ 50 × 10^9^/L] [8].

Early mortality was defined as a patient's death within the first year from the time of ALL diagnosis.

### 2.4. Detection of Gene Rearrangements

The identification of gene rearrangements was carried out at the Unidad de Investigación Médica en Inmunología e Infectología, Hospital de Infectología “Dr. Daniel Méndez Hernández,” “La Raza” from the IMSS.

#### 2.4.1. Cell Lines

In this study, cell lines were obtained from the American Type Culture Collection (ATCC) and used as positive controls for RT-PCR: the t(12; 21) positive cell line Reh (ATCC CRL-8286) (having the* ETV6/RUNX1* fusion gene); SUP-B15 (ATCC CRL-1929) (having t(9;22)(q34;q11), t(4;14) (p11;q24), der(4)t(1;4) (p11;q33), t(9;22) (q34;q11), der(10)t(3;10) (q25;q26), and (16); Philadelphia chromosome is present); RS4;11 (ATCC CRL-1873) (with t(4;11)(q21;q23); K-562 (ATCC CCL-243); p210^Bcr-Abl^ p185^Bcr-Abl^ positive cell line for* BCR-ABL major and minor*, respectively. B1H1 (ATCC 59171) HL-60 promyelocytic leukemia was used as a negative control. The t(1;19) (q23;p13) positive cell line 697 was obtained from DSMZ-German collection of microorganisms and cell cultures (Braunschweig, Germany). All cell lines were cultured in RPMI 1640 medium supplemented with 10% fetal calf serum and antibiotics (Invitrogen, Carlsbad, CA) and maintained according to the manufacturer's instructions. The total RNA was extracted from cell lines and used as internal controls (positive and negative).

#### 2.4.2. RNA Isolation and Synthesis of Complementary DNA (cDNA)

Total RNA was extracted from leukemic and normal cells using TRIzol reagent (Invitrogen, Carlsbad, CA) according to the manufacturer's instructions. The quality of RNA was confirmed by the presence of intact ribosomal RNA (28s and 18s bands) by denaturing agarose gel electrophoresis and visualized by UV illumination. A total of 3 *μ*g of total RNA was reverse-transcribed using the Superscript one-step RT-PCR with Platinum Taq Kit (Invitrogen Life Technologies, Carlsbad, CA, USA). The cDNA was incubated at 94°C for 10 min to inactivate the reverse transcriptase. Finally, the synthesized cDNA was stored at −20°C until use.

#### 2.4.3. Detection of Gene Rearrangements by Polymerase Chain Reaction (PCR)

Gene rearrangements were detected with a conventional RT-PCR assay as described in Daniel-Cravioto et al. [6] in a previous study. In addition, a commercial Multiplex RT-PCR kit was used according to the manufacturer's instructions (Hemavision, DNA-Technology A/S, Aarhus, Denmark). This screening assay covering 28 different fusion transcripts was used to test for the presence of more than 80 fusion transcript variants. After cDNA synthesis, the PCR amplification was performed in two steps: first, a master PCR amplification followed by nested PCR to screen for the presence of fusion transcripts and second a split-out PCR amplification followed by nested PCR to identify specific fusion transcripts [9, 10]. We obtained one hundred percent concordance between our in-house procedure and the commercially available kit. Because we were only interested in the four rearrangements, we decided, once validated, to use the conventional RT-PCR assay as described in Daniel-Cravioto et al. [6].

## 3. Statistical Analysis

Statistical analyses were performed using SPSS IBM (Statistical Package for the Social Sciences, Inc., Version 21, Chicago, IL, USA) and relative frequencies regarding the four most frequent gene rearrangements were obtained.

## 4. Results

From 2010 to 2012, the MIGICCL registered 638 pediatric patients newly diagnosed with ALL from 8 public hospitals of Mexico City of which 334 (52.4%) were male. The entire population median age was 6.2 years (75 months; range from 2 to 222 months). According to the NCI risk classification, 356 patients (55.8%) were classified as standard risk and 282 (44.2%) as high risk. The median WBC at diagnosis was 10.07 × 10^9^/L (range from 0.46 to 970 × 10^9^/L). According to their immunophenotype, 543 (85.1%) patients were classified as having B-cell precursor ALL, 69 (10.8%) patients as having the T-cell immunophenotype, and 26 (4.1%) patients as having biphenotypic leukemia. Early mortality occurred in 86 (13.5%) patients in our cohort study.

The analysis to detect the most frequent gene rearrangements was carried out on 282 (44.2%) available samples (Figure 1), and gene rearrangements were detected in 50 (17.7%) from these patients. Patient characteristics according to molecular subgroups are displayed in Table 1.

*ETV6-RUNX1* was detected in 21 patients (7.4%), of whom 14 (66.7%) patients were female. The* ETV6-RUNX1* gene rearrangement was observed in 18 (85.7%) children under the age of 10 and B-cell precursor was the predominant immunophenotype (Table 1). Moreover, the majority of patients (66.7%) had a standard risk and early mortality occurred in 1 (4.8%) patient with this fusion gene.

The* TCF3-PBX1* translocation was present in 20 (7.1%) patients. The B-cell precursor immunophenotype was observed in 19 (95%) patients and 1 patient (5%) had biphenotypic leukemia. In addition, ten patients (50%) with this rearrangement were classified as high risk and 1 (5%) patient died.

The frequency of patients with the* MLL* rearrangements was 1.4% (*n* = 4) of which 3 (75%) were female. Three patients presented the* MLL-AF4* rearrangement, and one case presented the* MLL-AF17* rearrangement. The patients' age ranged between 3 and 137 months with a median of 43 months (3.5 years). These patients had a median WBC of 210.7 × 10^9^/L (range from 17.4 to 970 × 10^9^/L) and 75% (*n* = 3) of* MLL* cases were classified as high risk patients. One of the four patients (25%) presented early mortality (Table 1).

The* BCR-ABL1* gene rearrangement was detected in five (1.8%) patients, four (80%) were male. The median age at diagnosis was 102 months (range from 15 to 185). Four (80%) patients had a B-cell precursor immunophenotype, and in this molecular subgroup early death occurred in two patients (40%).

Interestingly, the earliest deaths occurred at months 1, 2, and 3 after diagnosis in patients with the* MLL, ETV6-RUNX1,* and* BCR-ABL1* gene rearrangements, respectively.

## 5. Discussion

This is the first report regarding the prevalence of the four most frequent gene rearrangements in pediatric patients with ALL from eight public hospitals where ~69.9% of the all Mexico City's children with leukemia are treated [2].

### 5.1. *ETV6-RUNX1* Gene Rearrangement

The frequency of* ETV6-RUNX1* in the present study (7.4%) is consistent with reports from developing countries. In a previous study from 26 pediatric cases from the Hospital La Raza in Mexico City, the frequency was 3.8% [6]. In India [14] and Brazil [15], reported frequencies were 7% and 11.3%, respectively (Table 2). However, we observed a lower frequency of the* ETV6-RUNX1* rearrangement in comparison to reports from developed countries [11–13, 20]. Interestingly, the disparities regarding gene rearrangement prevalence among countries could be explained by environmental factors as playing an important role in the development of childhood leukemia, as would be the case of Mexico [1, 6].

Interestingly,* ETV6-RUNX1* patients with a B-cell precursor immunophenotype were predominantly female. This finding is consistent with previous reports [21, 22]. Notably, in our study, the majority of* ETV6-RUNX1* cases were under the age of 10. It has been reported that* ETV6-RUNX1* translocation is most commonly observed in children with leukemia under this age [23].

### 5.2. *TCF3-PBX1* Gene Rearrangement

The proportion of positive patients for the* TCF3-PBX1* gene rearrangement represents one of the highest reported worldwide [14–19, 24] (Table 2).Our findings agree with results reported by Jiménez-Morales et al. [5] who found that the frequency of* TCF3-PBX1* was 11.5% in children treated at a single institution in Mexico City. In our cohort, the proportion of* TCF3-PBX1* is intermediate with respect to those reported for white children (3.0%) and black children (11.8%) [16]. In addition, it has been reported that the* TCF3-PBX1* fusion gene is associated with a poor outcome in ALL patients [25, 26]. Therefore, the high frequency of* TCF3-PBX1* could possibly explain the high mortality rates observed in Mexican children with ALL.

### 5.3. *MLL* Rearrangements

With regards to the translocations involving the chromosome 11q23 gene* MLL*, a lower frequency was observed (1.4%) compared to that reported by Daniel-Cravioto et al. [6] at the Hospital La Raza of Mexico City (65.4%). However, our findings are consistent with the reported in developed countries (Table 2). The results reported in the work by Daniel-Cravioto et al. [6] regarding the frequency of this gene rearrangement could be explained by an event called random variability, which is very probable when frequencies of extremely rare diseases are reported. For example, in 1996, the incidence of childhood leukemia corresponded to 80 cases per million in Mexico City, and this value was an exceptional and astonishing observation that has not registered again [27]. On the other hand, the median age of the* MLL* rearrangements subgroup was 42 months, and two patients were less than 1 year of age. These findings are consistent with the literature surmising that this type of gene rearrangement is associated with leukemia in early life [28].

### 5.4. *BCR-ABL1* Gene Rearrangement

We observed a relatively low frequency (1.8%) of the* BCR-ABL1* gene rearrangement in comparison to previously reported studies carried out in Mexico City (Table 2). In addition, our findings are similar to those reported in Brazil (1.1%) [15] and Argentina (1.6%) [19]. Interestingly, two* BCR-ABL1* cases died during the first year of treatment. This gene rearrangement confers a poor prognosis in ALL patients [24] unless treated with tyrosine kinase inhibitors [TKIs] or hematopoietic stem cell transplant [HSCT]) [26].

### 5.5. Early Mortality during the First Year of Treatment

In our cohort (*n* = 638), the mortality rate during the first year of treatment was high (13.5%) considering that 5-year survival rates in developed countries, currently, are higher than 90% [29]. Early mortality was observed in patients with positive gene rearrangements too. In a study conducted by Curado et al. [3] they reported that Mexico is among the few countries worldwide that has failed to reduce the mortality caused by childhood leukemia. The authors also mentioned that in recent years there has been a significant increase in mortality rates for childhood leukemia in this country [3]. Children who are classified as having a lower risk are treated with less intensive chemotherapy, while those classified as having a higher risk might be given a higher dose of chemotherapy than required leading to a higher rate of toxicity and higher mortality rates [26]. The inclusion of gene rearrangement detection in risk-adapted therapy has significantly contributed to improve the survival rate of pediatric patients with ALL in developed countries [25, 30]. However, in the majority of Mexico City public hospitals, current risk allocation schemes do not include the detection of gene rearrangements [7]. This might explain why Mexican children with ALL have higher mortality rates compared to developed countries where molecular techniques are routinely carried out in all patients with leukemia [24]. However, to draw solid conclusions about the impact of gene rearrangements on the survival of Mexican children with ALL, a long-term followup is necessary.

## 6. Conclusions

We present results from a multicenter study carried out by the MIGICCL on the frequency of the four most common gene rearrangements in Mexican pediatric ALL patients. We found a particularly high prevalence of the* TCF3-PBX1* gene rearrangement (associated with a poor prognosis) and a low prevalence of the* ETV6-RUNX1* fusion gene (associated with a better prognosis) in comparison to developed countries [25, 31]. Studies on the frequency of gene rearrangements in children with ALL are very heterogeneous; this is most likely due to ethnic and/or environmental factors. Moreover, this is a significant finding, as it may help to explain the high mortality rates observed in Mexican children with ALL; however, further research is needed to elucidate this hypothesis. We recommend the routine detection of gene rearrangements at ALL diagnosis in Mexican children to provide a better prognostic stratification and help to decide the most appropriate treatment, as already implemented in developed countries.

## Figures and Tables

**Table 1:** Patient characteristics in molecular subgroups of childhood acute lymphoblastic leukemia (ALL). Results from the Mexican Interinstitutional Group for the Identification of the Causes of Childhood Leukemia (MIGICCL).

Patient characteristics	Gene rearrangement					Samples not available for screening (n = 356)
	*ETV6-RUNX1* (n = 21)	*TCF3-PBX1* (n = 20)	*BCR-ABL1* (n = 5)	*MLL * rearrangements (n = 4)	Undetected (n = 232)	
	n (%)	n (%)	n (%)	n (%)	n (%)	n (%)
Gender						
Male	7 (23.3)	10 (50)	4 (80)	1 (25)	115 (49.5)	197 (55.3)
Female	14 (66.7)	10 (50)	1 (20)	3 (75)	117 (50.4)	159 (44.7)
Age (in months)						
Range	31–144	9–187	15–185	3–137	5–222	2–203
Median age	75	95.5	102	43	84.5	68.5
Age groups						
<1 year	—	1 (5)	—	2 (50)	4 (1.7)	14 (3.9)
1–9 years	18 (85.7)	12 (60)	3 (60)	1 (25)	158 (68.1)	237 (66.6)
10–14 years	3 (14.3)	5 (25)	1 (20)	1 (25)	51 (22)	78 (21.9)
≥15 years	—	2 (10)	1 (20)	—	19 (8.2)	27 (7.6)
WBC count ×10^9^/L						
Range	2.2–306	0.9–137.6	4.6–149.5	17.4–970	0.4–910	0.6–697.8
Median	15.8	21.2	31.2	210.7	10.8	9.0
Immunophenotype						
B-cell precursor	20 (95.2)	19 (95)	4 (80)	3 (75)	205 (88.4)	292 (82)
T-cell	—	—	—	1 (25)	24 (10.3)	44 (12.4)
Biphenotypic	1 (4.8)	1 (5)	1 (20)	—	3 (1.3)	20 (5.6)
NCI risk classification						
Standard	14 (66.7)	10 (50)	3 (60)	1 (25)	121 (52.2)	207 (58.1)
High	7 (33.3)	10 (50)	2 (40)	3 (75)	111 (47.8)	149 (41.9)
Early death (1st year)						
Yes	1 (4.8)	1 (5)	2 (40)	1 (25)	31 (13.4)	50 (14)
No	20 (95.2)	19 (95)	3 (60)	3 (75)	201 (86.6)	306 (86)

**Table 2:** Proportions of the four molecular subgroups reported in the present study, other Mexican studies, and studies from other countries.

Author, year of publication [Reference]	Country	Number of patients	*ETV6-RUNX1* *n*(%)	*TCF3-PBX1* *n* (%)	*MLL* rearrangements *n* (%)	*BCR-ABL1* *n* (%)
MIGICCL study, 2014 [present study]	Mexico City	287	24 (7.4)	20 (7.1)	4 (1.4)	5 (1.8)
Other Mexican studies						
Pérez-Vera et al., 2008 [4]	Mexico City	59	5 (8.7)	—	5 (8.7)	1 (1.7)
Jiménez-Morales et al., 2008 [5]	Mexico City	53	7 (13.5)	6 (11.5)	—	2 (3.8)
Daniel-Cravioto et al., 2009 [6]	Mexico City	26	1 (3.8)	—	17 (65.4)	1 (3.8)
Studies from other countries						
Amor et al., 1998 [11]	Australia	66	22 (33.0)	—	—	—
Zuna et al., 1999 [12]	Czech Republic	190	41 (21.6)	—	—	—
Codrington et al., 2000 [13]	England	56	22 (39.0)	—	—	—
Siraj et al., 2003 [14]	India	259	18 (7.0)	18 (7.0)	2 (4)∗	14 (5.0)
Mesquita et al., 2003 [15]	Brazil	88	10 (11.4)	5 (5.7)	1 (1.1)	1 (1.1)
Pui et al., 2003 [16]						
White children	USA	338	64 (18.9)	10 (3.0)	10 (3.0)	8 (2.4)
Black children	USA	68	9 (13.2)	8 (11.8)	1 (1.5)	4 (5.9)
Aldrich et al., 2006 [17]						
Non-Hispanic White	USA	140	34 (24.3)	6 (4.3)	4 (2.9)	1 (0.7)
Hispanic	USA	151	19 (12.6)	7 (4.6)	3 (2.0)	2 (1.3)
Lazic et al., 2010 [18]	Serbia	70	12 (17.1)	6 (8.6)	0	7 (10.0)
Alonso et al., 2012 [19]	Argentina	380	49 (12.9)	19 (5.0)	40 (10.5)	6 (1.6)

MIGICCL: Mexican Interinstitutional Group for the Identification of the Causes of Childhood Leukemia.

— Not screened.

∗Other *MLL* rearrangements were detected in two (4%) of 50 samples analyzed by Southern blot.
